# Exosomes derived from bone-marrow mesenchymal stem cells alleviate cognitive decline in AD-like mice by improving BDNF-related neuropathology

**DOI:** 10.1186/s12974-022-02393-2

**Published:** 2022-02-07

**Authors:** Sen Liu, Min Fan, Jing-Xian Xu, Long-Jun Yang, Cong-Cong Qi, Qing-Rong Xia, Jin-Fang Ge

**Affiliations:** 1 School of Pharmacy, Anhui Medical University, 81 Meishan Road, Anhui 230032 Hefei, People’s Republic of China; 2 The Key Laboratory of Anti-Inflammatory and Immune Medicine, Ministry of Education, Anhui Medical University, Hefei, China; 3 Anhui Provincial Laboratory of Inflammatory and Immunity Disease, Anhui Institute of Innovative Drugs, Hefei, China; 4 Chaohu Clinical Medical College, Anhui Medical University, Hefei, China; 5 Neurodevelopmental Laboratory, Fudan University, Shanghai, China; 6 Department of Pharmacy, Hefei Fourth People’s Hospital, Anhui Mental Health Center, 316 Huangshan Road, Hefei, 230032 China; 7 Psychopharmacology Research Laboratory, Anhui Mental Health Center, Hefei, China; 8 Clinical Pharmacy, Affiliated Psychological Hospital of Anhui Medical University, Hefei, China

**Keywords:** Alzheimer's disease, Bone-marrow stem cell, Exosome, Neuroinflammation, Synaptic plasticity

## Abstract

**Background:**

Alzheimer's disease (AD) is a neurodegenerative disease characterized by a progressive decline in cognitive ability. Exosomes derived from bone-marrow mesenchymal stem cells (BMSC-exos) are extracellular vesicles that can execute the function of bone-marrow mesenchymal stem cells (BMSCs). Given the versatile therapeutic potential of BMSC and BMSC-exos, especially their neuroprotective effect, the aim of this study was to investigate the potential effect of BMSC-exos on AD-like behavioral dysfunction in mice and explore the possible molecular mechanism.

**Methods:**

BMSC-exos were extracted from the supernatant of cultured mouse BMSCs, which were isolated from the femur and tibia of adult C57BL/6 mice, purified and sorted via flow cytometry, and cultured in vitro. BMSC-exos were identified via transmission electron microscopy, and typical marker proteins of exosomes were also detected via Western blot. A sporadic AD mouse model was established by intracerebroventricular injection of streptozotocin (STZ). Six weeks later, BMSC-exos were administered via lateral ventricle injection or caudal vein injection lasting five consecutive days, and the control mice were intracerebroventricularly administered an equal volume of solvent. Behavioral performance was observed via the open field test (OFT), elevated plus maze test (EPM), novel object recognition test (NOR), Y maze test (Y-maze), and tail suspension test (TST). The mRNA and protein expression levels of IL-1β, IL-6, and TNF-α in the hippocampus were measured via quantitative polymerase chain reaction (qPCR) and Western blot, respectively. Moreover, the protein expression of Aβ_1-42_, BACE, IL-1β, IL-6, TNF-α, GFAP, p-Tau (Ser396), Tau5, synaptotagmin-1 (Syt-1), synapsin-1, and brain-derived neurotrophic factor (BDNF) in the hippocampus was detected using Western blot, and the expression of GFAP, IBA1, Aβ_1−42_ and DCX in the hippocampus was measured via immunofluorescence staining.

**Results:**

Lateral ventricle administration, but not caudal vein injection of BMSC-exos improved AD-like behaviors in the STZ-injected mouse model, as indicated by the increased number of rearing, increased frequency to the central area, and increased duration and distance traveled in the central area in the OFT, and improved preference index of the novel object in the NOR. Moreover, the hyperactivation of microglia and astrocytes in the hippocampus of the model mice was inhibited after treatment with BMSC-exos via lateral ventricle administration, accompanied by the reduced expression of IL-1β, IL-6, TNF-α, Aβ_1-42,_ and p-Tau and upregulated protein expression of synapse-related proteins and BDNF. Furthermore, the results of the Pearson test showed that the preference index of the novel object in the NOR was positively correlated with the hippocampal expression of BDNF, but negatively correlated with the expression of GFAP, IBA1, and IL-1β. Apart from a positive correlation between the hippocampal expression of BDNF and Syt-1, BDNF abundance was found to be negatively correlated with markers of glial activation and the expression of the inflammatory cytokines, Aβ_1-42_, and p-Tau, which are characteristic neuropathological features of AD.

**Conclusions:**

Lateral ventricle administration, but not caudal vein injection of BMSC-exos, can improve AD-like behavioral performance in STZ-injected mice, the mechanism of which might be involved in the regulation of glial activation and its associated neuroinflammation and BDNF-related neuropathological changes in the hippocampus.

**Supplementary Information:**

The online version contains supplementary material available at 10.1186/s12974-022-02393-2.

## Background

Alzheimer's disease (AD) is a neurodegenerative disease characterized by progressive cognitive decline, accounting for approximately 50–60% of all dementias [1]. Sporadic AD is the most notable form of AD, with complex pathogenesis accounting for more than 90% of all clinical cases of AD [2, 3]. According to the latest statistics from the Alzheimer’s Association of the United States, as of 2020, more than 50 million people worldwide have suffered from dementia, and this number will reach 150 million by the middle half of the twenty-first century. At present, the global cost of dementia has reached 818 billion dollars, and a case of dementia occurs about every 3 s worldwide [4]. Although investigations targeting the pathogenesis and treatment of AD have been ongoing for more than a century, the etiology of AD is still controversial [5]. Accordingly, “Brain Plan”, the targeted investigation into the pathogenesis of AD and potential therapeutic drugs, has been carried out in many countries.

The main and more established hypotheses are the amyloid and tau hypotheses, which have been highly debated over the last decades. It has been demonstrated that the overproduction of amyloid β peptide could result in the hyperphosphorylation of Tau, which together may lead to altered synaptic function, increased inflammatory responses and oxidative injury, and altered kinase and phosphatase activities, eventually resulting in the deposition of amyloid plaques and neurofibrillary tangle formation. However, a considerable number of clinical trials of anti-AD drugs focusing on amyloid and neurofibrillary tangle formation, have mostly ended in failure [6]. More recently, the U.S. Food and Drug Administration (FDA) granted accelerated approval for aducanumab, which is the first new drug for AD targeting at amyloid β. However, its effect remains controversial [7–10], and it is suggested by the FDA that a new randomized controlled clinical trial should be carried out to examine the clinical benefits of the drug [11]. Thus, it is still needed to investigate thoroughly the pathogenesis of AD and to explore potential therapeutic methods.

Exosomes are extracellular vesicles with a diameter of approximately 30 ~ 200 nm that carry important genetic information, such as mRNAs, microRNAs (miRNAs) and proteins. Information transmission and communication between cells can be mediated by exosomes [12]. Compared with that in the normal population, the abundance and miRNA profiles of exosomes were significantly changed in the plasma and cerebrospinal fluid of patients with neuropsychiatric diseases, including AD and depression [13–15]. In line with these findings, the results of our previous studies have demonstrated a different miRNA expression profile of serum exosomes in depression-like and the control samples [16], suggesting that exosomes and their contents play different roles under different physiological conditions.

Bone-marrow mesenchymal stem cells (BMSCs) are typical stem cells that can be differentiated into different cells under different physiological conditions, and they can selectively migrate to the site of tissue damage, interact with brain cells, and then stimulate the production of growth factors such as brain-derived neurotrophic factor (BDNF) and nerve growth factor (NGF) [17–19]. BMSCs are considered to be the most effective exosome-producing cells [20]. Compared with BMSCs themselves, exosomes derived from BMSCs (BMSC-exos) have more advantages in that they will not replicate or cause proliferation, thus there is no risk of inducing tumor formation, and they will not undergo metabolism, not affect the environment, and not be affected by the environment, either. Most importantly, BMSC-exos have similar effects as BMSCs but without the side effects [21–23]. There are many in-depth and extensive studies investigating the biological functions of BMSCs and BMSC-exos, demonstrating their beneficial effects against heart diseases [24, 25], kidney injuries [26], ulcers [27], and so on. For example, exosomal miR-486 derived from adipose-derived stem cells can be used as an autophagy activator and transferred into podocytes to reduce the cell damage of mouse renal podocytes (MPC5) in diabetic mice [26]. Dittmer et al. found great advantages of mesenchymal stem cells in promoting ulcer wound healing, while exosomes released by mesenchymal stem cells and microvesicles were reported to play a key role in wound healing [27]. More recently, the therapeutic effect of mesenchymal stem cell exosomes against neuropsychiatric diseases has also been demonstrated. It has been reported that neuroinflammation and neuronal apoptosis in primary neurons of transgenic AD mice could be reduced by treatment with exosomes derived from mesenchymal stem cells [28]. Moreover, Long et al. found that exosomes derived from mesenchymal stem cells administered intranasally could reduce inflammation and prevent abnormal neurogenesis and memory dysfunction after status epilepticus, but its mechanism of action needs to be further elucidated [29]. However, the effect of BMSC-exos on neuroinflammation and cognitive decline in AD animal models has not yet been elucidated.

In the present study, to investigate the potential effect of BMSC-exos against sporadic AD-like behavior, a sporadic AD mouse model was established by intracerebroventricular injection of streptozotocin (STZ), and BMSC-exos were administered 6 weeks later via lateral ventricle injection or caudal vein injection. Behavioral performance was observed by the open field test (OFT), elevated plus maze test (EPM), novel object recognition test (NOR), Y maze test (Y-maze), and tail suspension test (TST). Nissl staining was used to confirm the injection site of exosomes. Glial cell activation, new neurons and the positive area of Aβ_1-42_ in the hippocampus were observed by immunofluorescence staining (IF). The protein expression levels of IBA1, GFAP, IL-1β, IL-6, TNF-α, BACE, Aβ_1-42_, p-Tau, Tau5, synaptotagmin-1 (Syt-1), synapsin-1, and BDNF in the hippocampus were detected via Western blot, and the mRNA expression levels of IL-1β, IL-6 and TNF-α were measured by quantitative real-time PCR (qPCR).

## Materials and methods

### Cell culture medium

BMSC complete medium consisted of α-MEM basic medium (BL306A, Biosharp) plus 10% fetal bovine serum (04-001-1ACS, Biological Industries), 1% penicillin–streptomycin solution (C0222, Beyotime), 20 ng/mL EGF (50482-MNCH LC13JA1403, Sino Biological), and 20 ng/mL bFGF (50177-M08H LC12SE1702, Sino Biological).

### Isolation, culture and purification of primary BMSCs

Adult male C57BL/6 mice were sacrificed by cervical dislocation and then sterilized and immersed in 75% ethanol for 5–10 min. The femur and tibia were separated under aseptic conditions and cut into pieces with a volume less than 3 mm^3^. Red blood cells were lysed with red blood cell lysate (C3702-120 mL, Beyotime), and after centrifugation, the fragments were resuspended in culture medium, inoculated in a culture flask, and placed in an incubator at 37 °C, 5% CO_2_ and 95% O_2_ with saturated humidity. Cells were passaged each time the cells formed a certain number of colonies in the culture flask. Third-generation cells incubated with APC-CD44 (103012, Biolegend), FITC-CD45 (103107, Biolegend) and PE-CD11b (101207, Biolegend) were used for flow sorting, and the CD44^+^/CD45^−^/CD11b^−^ cells were sorted by a Beckman flow cytometry sorting system (Moflo-XDP, Backman). The sorted cells were collected in a sterile flow tube, transferred to a cell culture flask to continue culturing, and the culture medium was replaced every 48 h.

### Extraction, separation, and fluorescent labeling of BMSC-exos

After the sorted cell culture reached a certain density, the complete culture medium was replaced with exosomal-free serum, and the cell supernatant was collected every 48 h. Exosomes were extracted using a cell supernatant exosome isolation kit (4478359, Thermo Fisher Scientific), resuspended in PBS, and frozen at − 20 °C for later use. The BCA protein quantification method was used to quantify the extracted exosomes, the expression of exosomal marker proteins was detected by Western blot, and the PKH26 staining kit (MX4021-100UL, Maokangbio) was used to stain and label the exosomes. In preparation for injection via the lateral ventricle or caudal vein, the exosomes were resuspended and collected with an appropriate amount of prechilled artificial cerebrospinal fluid (ACSF).

### Animals

Twenty-four male C57BL/6 mice aged 4 weeks were purchased from the Anhui Animal Center. The animals were bred in a 12 h alternating light and dark cycle environment with an ambient temperature of 20 ± 2 ℃ and humidity 50 ± 5%, and they had free access to food and water. When the mice grew to 12 weeks of age, they were randomly divided into a control group (6 animals) and an STZ group (18 animals), with three animals in each cage. All animal manipulation procedures were approved by the Experimental Animal Ethics Committee of Anhui Medical University and complied with the "Guidelines for the Care and Use of Laboratory Animals of the National Institutes of Health" (NIH Publication No. 85–23, revised in 1985).

### Establishment of animal model and experimental design

All mice were anesthetized by intraperitoneal injection of 5% chloral hydrate (0.1 mL/10 g) and fixed on the brain stereotaxic device. After the surface disinfection, and the scalp incised. Then the Bregma was located, the coordinates of the bilateral ventricles were drilled by an electric cranial drill and get two burr holes (the coordinates were 1.0 mm left and right of the Bregma, 0.5 mm back, and 2.5 mm deep). And then the STZ was injected into the lateral ventricle of mice by the autosampler in the STZ group (the injection dose was 0.3 mg/kg, the injection speed was 0.5 μL/min, the injection volume was 1 μL/side, and the STZ was dissolved in ACSF and prepared for current use). Mice in the control group were given ACSF at an equal volume. At the end of the injection, the needle was stopped for 5 min and then the needle was withdrawn. The mouse scalp was sutured, and an appropriate amount of normal saline was injected into the intraperitoneal cavity and then the mouse was transferred to an electric blanket until awakened and placed in a squirrel cage.

**Fig. 1:**
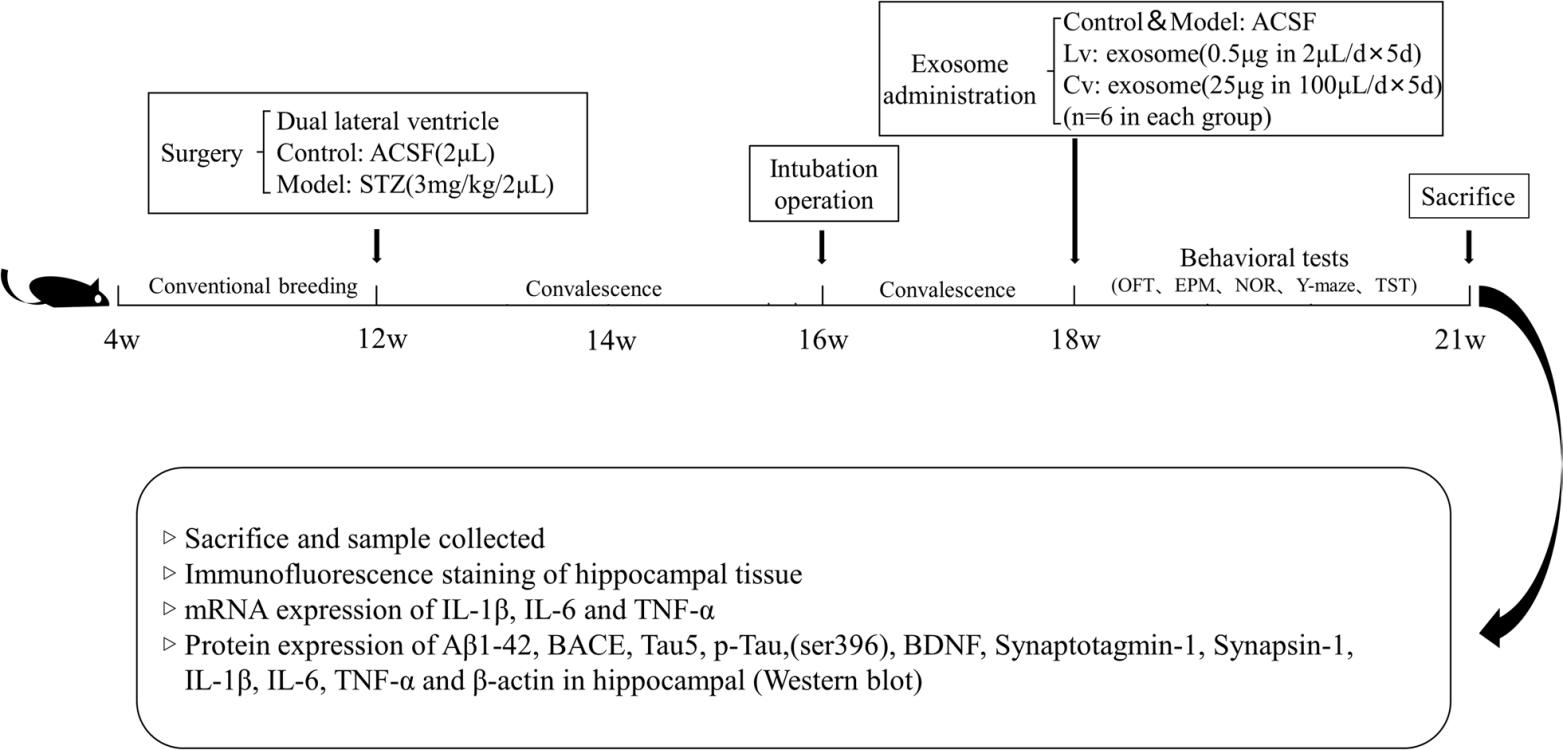
Experimental process. *STZ* streptozotocin, *ACSF* artificial cerebrospinal fluid, *OFT* open field test, *NOR* novel object recognition test, *Y-maze* Y maze test, *TST* tail suspension test

After a 1-month recovery period for all the mice, the STZ-injected mice were divided into 3 groups: a model group, an exosomal lateral ventricle injection (Lv) group, and an exosomal caudal vein injection (Cv) group, with 6 mice in each group. The right cerebral ventricle of all mice was intubated using a stereotaxic instrument and a drug delivery catheter (62003, 62102, RWD). The mice in the Lv group were injected with 0.5 μg BMSC-exos (dissolved in 2 μL ACSF) per day in the lateral ventricle, the control group and the model group were injected with equal volumes of ACSF, and the mice in the Cv group were injected with 25 μg BMSC-exos (dissolved in 100 μL PBS) per day in the tail vein. Administrations were performed on all mice for 5 days. After administration, a series of behavioral tests were performed on all mice. The experimental time and steps are shown in Fig. 1.

### Behavioral tests

All behavioral tests were performed in a sound-proof and quiet room with dim light from wall lamps scattered in the corner of the room, and during the light phase of the light/dark cycle. All mice were given 30 min to adapt to the environment before the behavioral experiments. All of the tests were carried out between 08:30 and 12:30, and they were matched between the groups. The observers were blind to the treatment. The behavioral tests were monitored and recorded by a digital camera interfaced to a computer running the ANY-maze video imaging software (Stoelting Co, Wood Dale, USA).

### Open field test (OFT)

The experimental apparatus of the OFT was a white cube opaque box 45 cm × 45 cm × 45 cm in size. Each mouse was placed in the box with its back facing one side of the box wall and allowed to explore freely for 5 min. The total ambulatory distance and the duration, frequency and distance in the center and the number of rearing were recorded.

### Elevated plus maze test (EPM)

The EPM apparatus consisted of two opposite open arms (50 × 10 × 0.5 cm), two opposite closed arms (50 × 10 × 20 cm), and a central open platform (10 × 10 cm). Each mouse was placed on the central platform facing one of the open arms and allowed to explore the instrument freely for 5 min. The moving distance of each mouse in the closed arm was counted.

### Y maze test (Y-maze)

The Y-maze apparatus was composed of three white opaque plexiglass arms. The size of each arm was 40 cm × 20 cm × 10 cm. This task consisted of 2 sessions (training and test) and was conducted on 2 successive days. In the training session, the novel arm was closed, and the mice were placed at the end of the starting arm facing the wall and allowed to explore in the maze for 10 min. Twenty-four hours later, the test session was performed. The novel arm was opened, and the mice were permitted to explore the three arms for 5 min. The ambulatory distance in each arm was recorded, and the ratio of moving distance in the novel arm to that in the total arm was taken as the preference index of the novel arm.

### Novel object recognition test (NOR)

The NOR apparatus was a white cube opaque box 45 cm × 45 cm × 45 cm in size. The experiment was divided into two stages. The first three days were considered the first stage (adaptation period). Each mouse was placed in the apparatus facing one sidewall every day and allowed to explore and adapt freely for 10 min. The fourth day was the second stage (the inspection period). The inspection period was divided into two stages. In the first stage, two objects of the same shape, size, and color were placed on the two thirds of the diagonal of the bottom of the box. Each mouse was placed inside the instrument facing one side, allowed to explore freely for 10 min, and entered the second stage after 1 h. In the second stage, one of the objects was replaced by a novel object with different shapes and colors, and each mouse was put back into the apparatus and allowed to explore freely for 5 min. Behavioral software was used to record the mouse's behavior toward the novel and familiar objects for 5 min. The mouse's preference for novel and familiar objects was calculated, that is, the novel object preference index: time spent exploring novel objects/total time to explore novel and familiar objects, and statistical analysis was perform.

### Tail suspension test (TST)

The TST apparatus was a 40 cm high white open box. A 1.5 cm section of the tail end of the mouse was fixed with paper tape and hung on a crossbar directly above the box. The experimental process was recorded by an automatic camera. Each mouse was suspended by its tail for 6 min. The first 2 min was the adaptation period, and the last 4 min was the official tail suspension time. The immobility time of the mice in the last 4 min was observed and recorded with a video camera. The researcher made a judgement based on keeping the two front paws of the mouse as the standard, and all the results were recorded by the same person.

### Transmission electron microscopy observation of BMSC-exo morphology

A small amount of exosomal suspension was placed on parafilm, and the exosomes in the suspension were absorbed with sample-loaded copper mesh for 3 min. Then, the cells were stained with 2% phosphotungstic acid negative staining solution for 3 min. Finally, the sample was transferred to a transmission electron microscope for observation and image collection.

### Immunofluorescence staining (IF)

Three mice in each group were randomly selected for cardiac perfusion, perfused with PBS until there was no more blood outflow, replaced with 4% paraformaldehyde perfusion, and reperfused for 3 min after the systemic spasm. The whole brain of each mouse was removed completely and fixed overnight in 4% paraformaldehyde solution at room temperature, immersed in 30% sucrose solution and dehydrated to the bottom. The whole brain was placed inside a disposable embedding box, filled with OCT embedding solution and frozen at – 80 ℃ overnight. Slices with 30 μm thick were prepared with a cryostat, attached to a glass slide, returned to room temperature and dried in a fume hood for 10 min. The tissue was demarcated with an immunohistochemistry pen, and goat serum (ZLI-9022, ZSGB-BIO) was used to seal the sections at room temperature for 30 min. After the liquid was removed, the sections were incubated with diluted rabbit anti-GFAP (1:100, 16825-1-AP, Proteintech), rabbit anti-DCX (1:100, 13925-1-AP, Proteintech), mouse anti-Aβ (1:100, 800712, Biolegend), rabbit anti-p-Tau(Ser396) (1:100, WL03540, Wanleibio) or rabbit anti-Iba-1 (1:100, DF6442, Affinity) overnight at 4 °C in the dark. After washing with PBST, the sections were incubated with goat anti-rabbit FITC or goat anti-rabbit Cy3 at 37 °C for 1 h. After washing with PBST, the plates were sealed with anti-fluorescence quenching mounting tablets containing DAPI (S2110, Solarbio), and then the plates were mounted on a laser scanning confocal fluorescence microscope (LSM800, ZEISS) for imaging.

### Nissl staining

The slices of the ventricles were degreased with xylene, stained with Nissl solution to dark blue, and washed with double distilled water, and an appropriate amount of differentiation solution was added dropwise for differentiation. Then, the sections were dehydrated with gradient alcohol, degreased again with xylene, and finally sealed with neutral gum. The slices were placed in a fume hood to dry for 2 h, and then images were observed and collected with a microscope.

### Thioflavin S staining

The sections were fixed with 95% ethanol and then covered with 1% Thioflavin S for 1 h. After the slices were differentiated with 50% ethanol, the images were collected under a fluorescence microscope.

### Western blot assays

#### Detection of the exosomal marker proteins

The exosomal suspension was mixed with SDS protein loading buffer for Western blot experiments to detect the expression of the surface marker proteins anti-CD63 (1:1000; sc-5275, Santa Cruz), anti-TSG101 (1:1000; WL05130, Wanleibio), and anti-HSP70 (1:1000; WL01019, Wanleibio) expression, with the cell culture supernatant as a control.

#### Detection of protein expression in mouse hippocampus

In each group, 3 mice were selected immediately after cervical dislocation and sacrificed. The brains were dissected immediately, and hippocampal tissues were isolated and stored in liquid nitrogen. The Western blot method was performed as described in our previous research [30], using RIPA (P0013B, Beyotime) lysate containing protease inhibitors and phosphatase inhibitors to lyse the tissues and using the BCA protein quantification kit (P0010, Beyotime) to determine the protein concentration in each sample. The protein loading buffer and protein sample were mixed in boiling water and heated for 10 min. After cooling, the sample was added to a 12.5% SDS-PAGE gel. After electrophoresis, the membrane was transferred, and the protein was quickly blocked. The primary antibody was incubated overnight at 4 °C. The main antibody dilution ratios were as follows: anti-Aβ_1−42_ (1:1000; 25524-1-AP, Proteintech), anti-Tau5 (1:1000; sc-58860, Santa Cruz), anti-p-Tau (Ser396) (1:1000; sc-32275, Santa Cruz), anti-BACE (1:500; WL02795, Wanleibio), anti-GFAP (1:1000; 16825-1-AP, Proteintech), anti-synaptotagmin-1 (1:1000; YT4484, ImmunoWay), anti-synapsin-1 (1:1000; BS3667, Bioworld), anti-BDNF (1:1000; ab108319, Abcam), anti-IL-1β (1:500; WL00891, Wanleibio), anti-IL-6 (1:1000; WL02841, Wanleibio), anti-TNF-α (1:1000; WL01581, Wanleibio), and anti-β-actin (1:1000; TA-09, ZSGB-BIO). The membranes were then processed with appropriate HRP-conjugated secondary antibodies based on the proteins of interest. The protein levels were analyzed using ImageJ (Wayne Rasband, National Institutes of Health, USA) and normalized relative to that of the internal control β-actin.

### Quantitative real-time PCR (qPCR)

Quantitative real-time polymerase chain reaction (qPCR) was performed to determine IL-1β mRNA, IL-6 mRNA and TNF-α mRNA levels in all experimental groups. Part of the hippocampus from three mice in each group was used to extract RNA. The extraction steps were as follows: Trizol (15596026, Ambion) was used to lyse the tissues, chloroform was added, and the samples were incubated and then centrifuged. Then, the supernatant was mixed well with isopropanol and precipitated overnight at − 20 °C. The supernatant was discarded after centrifugation, and the precipitate was resuspended in 75% ethanol. The supernatant was discarded after centrifugation, the precipitate was resuspended in enzyme-free water, and RNA quantification was performed. After quantification was completed. Evo M-MLV RT Premix (AG11706, Accurate Biology) was used for reverse transcription, and cDNA, upstream primers, downstream primers, enzyme-free water and SYBR Green (AG11701, Accurate Biology) were used for real-time fluorescence quantification. PCR was performed in a thermal cycler as follows: 95 ℃ for 3 min, followed by 40 cycles of 95 ℃ for 10 s, and 55 ℃ for 30 s. The IL-1β-F primer sequence was 5′-CTTTGAAGTTGACGGACCC-3′, the IL-1β-R primer sequence was 5′-TGAGTGATACTGCCTGCCTG-3′, the IL-6-F primer sequence was 5′-AGTCCGGAGAGGAGACTTCA-3′, the IL-6-R primer sequence was 5′-ATTTCCACGATTTCCCAGAG-3′, the TNF-α-F primer sequence was 5′-CACCACCATCAAGGACTCAA-3′, the TNF-α-R primer sequence was 5′-AGGCAACCTGACCACTCTCC-3′, the β-actin-F primer sequence was 5′-AGTGTGACGTTGACATCCGT-3′, and the β-actin-R primer sequence was 5′-TGCTAGGAGCCAGAGCAGTA-3′. The experimental results were calculated using the 2^∆∆CT^ method and normalized analysis.

### Statistical analysis

All statistical analyses were performed using SPSS (Statistical Package for the Social Sciences, version 17.0). Data are expressed as the mean ± S.E.M., and *P* < 0.05 was considered statistically significant. Intergroup statistical analysis was performed by one-way analysis of variance (ANOVA) followed by the LSD post hoc test. All statistical charts were drawn using GraphPad Prism 7.0 and Microsoft PowerPoint 16.0.

## Results

### Sorting and cultivation of BMSCs

**Fig. 2:**
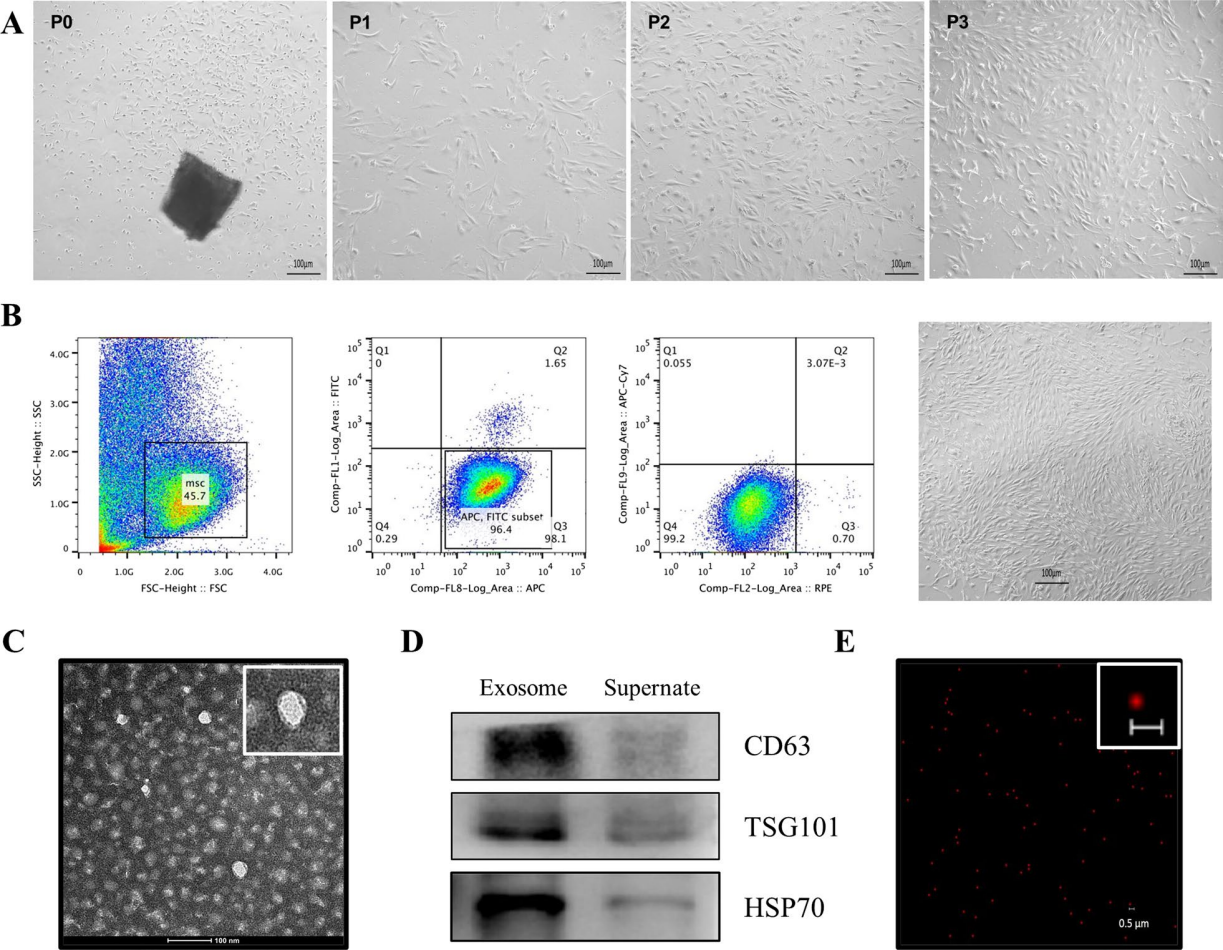
Extract, culture, and sorting of BMSCs, and identification and label of BMSC-exos. **A** Bone-marrow primary adherent cells before sorting (P0, Primary cells; P1, passage 1; P2, passage 2; P3, passage 3). **B** CD44^+^/CD45^−^ (96.4%) and CD11b^−^ (99.2%) BMSCs were sorted out by flow sorting, BMSCs grew into fibrous and colony-like growth, scale bar 100 μm. **C** Exosome morphology under transmission electron microscope. **D** Western blot photos of exosome markers. **E** Fluorescence imaging of exosome labeled with PKH26

As shown in Fig. 2A, after sorting and purification, BMSCs proliferated normally and grew in a fibrous and colony-like manner.

### Identification and fluorescent labeling of BMSC-exos

The CD44-positive (CD44^+^), CD45 and CD11b-negative (CD45^−^, CD11b^−^) cells were marked by flow cytometry, and CD44^+^/CD45^−^/CD11b^−^ BMSCs were sorted by a flow cytometry sorting system (Fig. 2B). BMSC-exos were extracted using a commercial kit. Under a transmission electron microscope, the BMSC-exos showed a typical double-layer membrane and cup holder-like structure with a particle size of approximately 50 nm (Fig. 2C). The exosomal marker proteins CD63, HSP70, and TSG101 were highly expressed (Fig. 2D). After staining with PKH26 reagent, a red circle could be found under a confocal microscope (Fig. 2E).

### Successful injection of exosomes into the ventricles and delivery to the hippocampus

**Fig. 3:**
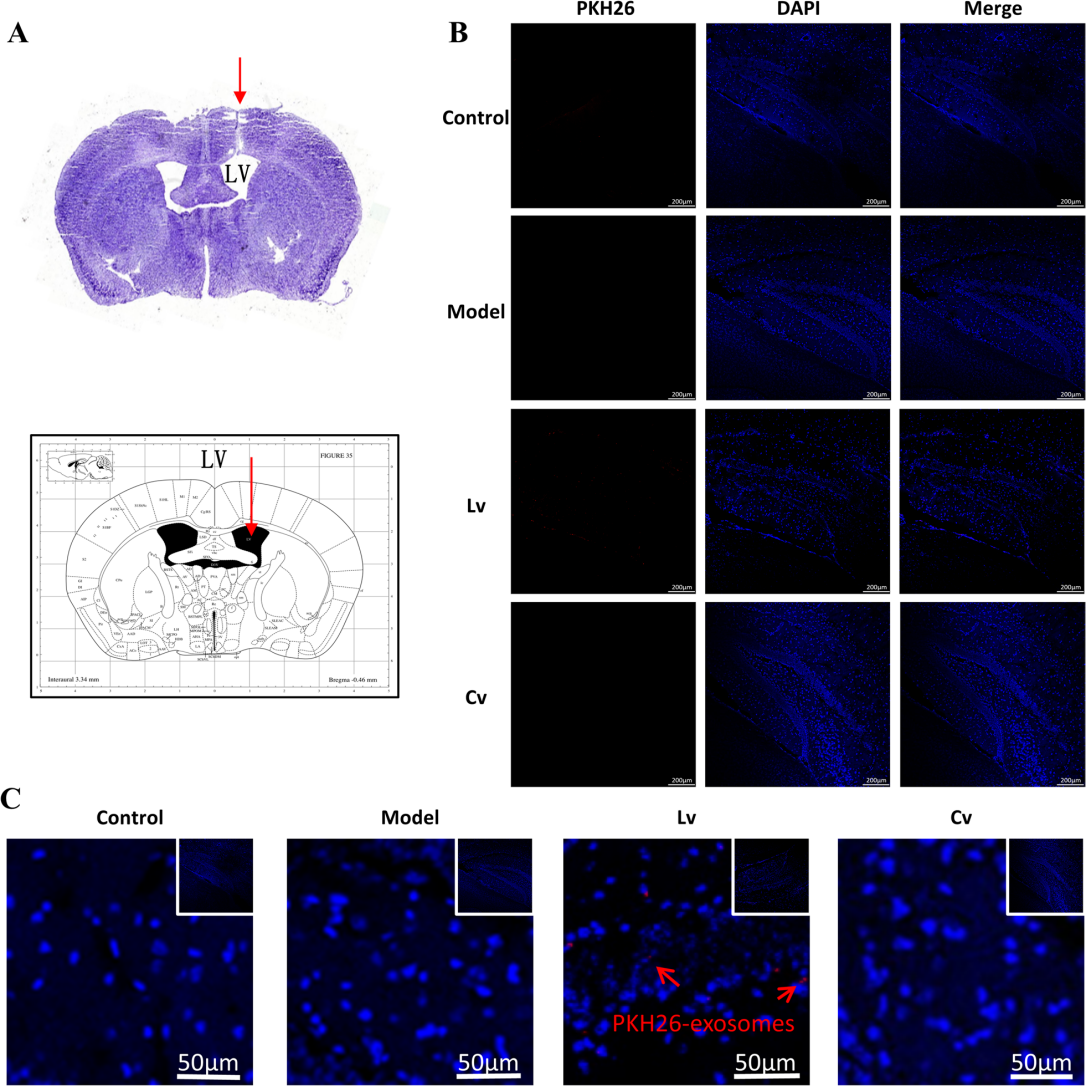
Exosomes injection site and its trace. **A** Nissl staining shows that the catheter is accurately positioned to the lateral ventricle, compare with the mouse brain map. **B** PKH26-labeled red fluorescent exosome particles are observed in the hippocampus of Lv group. **C** Partial enlarged view of exosomes labeled with PKH26

The results of Nissl staining showed that the delivery catheter was accurately positioned to the lateral ventricle, confirming the delivery location of BMSC-exos, which is shown in Fig. 3A. The results of IF showed that certain PKH26-labeled red exosomes could be detected in the hippocampus of the Lv group but not in the other groups, confirming that BMSC-exos can be delivered to the hippocampus, as shown in Fig. 3B, C.

### Lateral ventricle administration, but not caudal vein injection of BMSC-exos, improved the AD-like behaviors in the STZ-injected mouse model

**Fig. 4:**
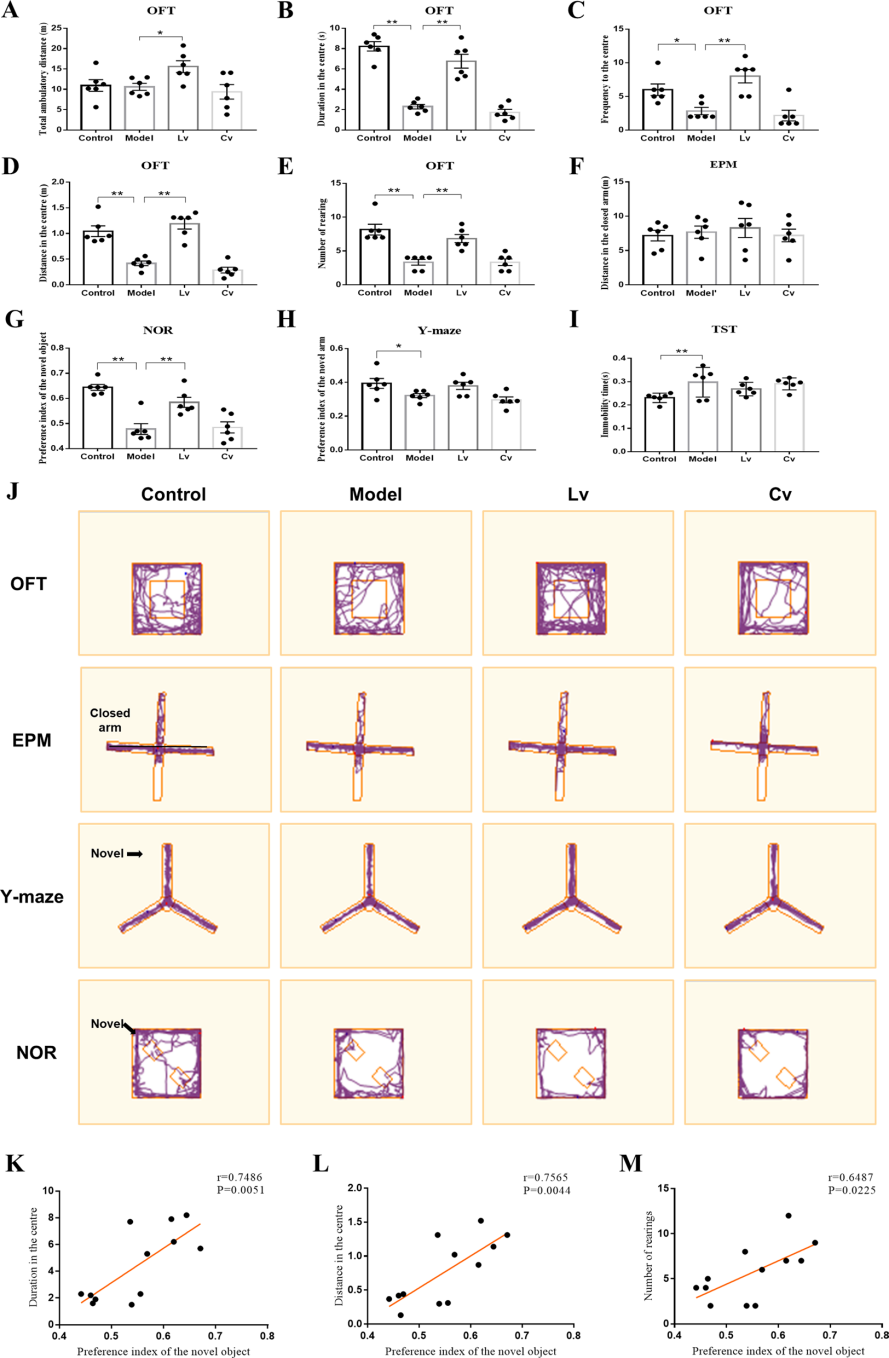
Lateral ventricle administration of BMSC-exos improved the AD-like behaviors induced by intracerebroventricular injection of STZ in mice. **A** Total ambulatory distance in the OFT. **B** Duration in the centre in the OFT. **C** Frequency to the central region in the OFT. **D** Moving distance in the central region in the OFT. **E** Number of rearing in the OFT. **F** Moving distance in the closed arm in the EPM. **G** Preference index of the novel object in the NOR. **H** Preference index of the novel arm in the Y-maze. **I** Immobility time in the TST. **J** Representative trajectories in OFT, EPM, Y-maze and NOR. **K** The duration in the centre in the OFT was positively correlated to the preference of novel object in the NOR. **L** The distance in the centre in the OFT was positively correlated to the preference of novel object in the NOR. **M** The number of rearing in the OFT was positively correlated to the preference of novel object in the NOR. Data are presented as means ± SEM, with *n* = 6 in each group (*P < 0.05, **P < 0.01)

Figure 4 shows the behavioral performance of all the mice in this study. The OFT is a common measure of exploratory behavior and general activity in rodents. Although there was no significant difference among groups regarding to the total ambulatory distance in the OFT (Fig. 4A), the STZ intracerebroventricularly injected model mice showed less duration (Fig. 4B), less frequency (Fig. 4C), less moving distance (Fig. 4D), and less rearing (Fig. 4E) in the central area than the control mice. These changes were reversed in the Lv group but not in the Cv group (Fig. 4B–D).

The EPM is a prominent and well-documented test for studying anxiety-like behaviors in rodents, and NOR and Y-maze tests are consistently used to evaluate learning and memory ability. As shown in Fig. 4, in the EPM test, there was no significant difference among the groups in the distance traveled in the closed arm (Fig. 4F). Compared with that of the control mice, the preference index of the novel object in the NOR test (Fig. 4G) and the preference index of the novel arm in the Y maze (Fig. 4H) of the model mice were both decreased, while the immobility time in the TST (Fig. 4I) was increased, which is an index of despair behavior. Lateral ventricle administration of BMSC-exos increased the preference index of the novel object in the NOR test (Fig. 4G), but had no significant effect on the preference index of the novel arm in the Y maze (Fig. 4H) or the immobility time in the TST (Fig. 4I). Typical trajectories of mice in the OFT, EPM, NOR, and Y-maze are shown in Fig. 4J.

The results of Pearson's correlation test showed that the preference for novel objects in the NOR was positively correlated with the duration in the central area in the OFT (*r* = 0.7486, *P* = 0.0051, Fig. 4K), distance in the center in the OFT (*r* = 0.7565, *P* = 0.0044, Fig. 4L) and the number of rearing in the OFT (*r* = 0.6487, *P* = 0.0225, Fig. 4M).

### Lateral ventricle administration, but not caudal vein injection of BMSC-exos, inhibited microglial activation and neuroinflammation in the hippocampus of the STZ-injected AD mouse model

**Fig. 5:**
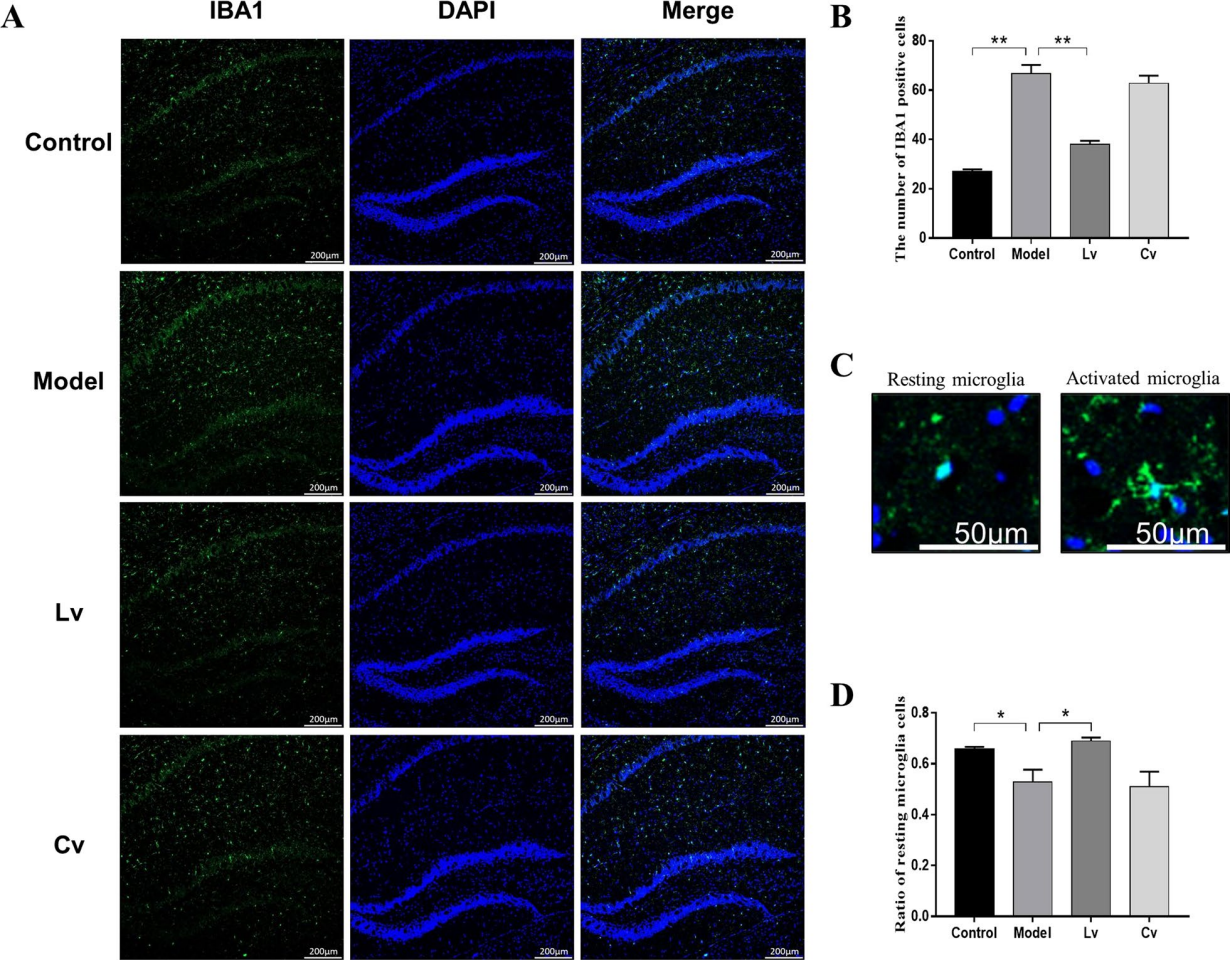
Lateral ventricle administration of BMSC-exos inhibited the proliferation and activation of microglia in the hippocampus of mice injected with STZ. **A** Fluorescence detection of microglia marker IBA1 in hippocampus **B** Quantification of the number of IBA1 positive cells. **C** Microglia in different states (left: resting state; right: activated state); **D** The ratio of activated microglia to the total number of microglia. Data are presented as means ± SEM, with *n* = 3 in each group (*P < 0.05, **P < 0.01)

**Fig. 6:**
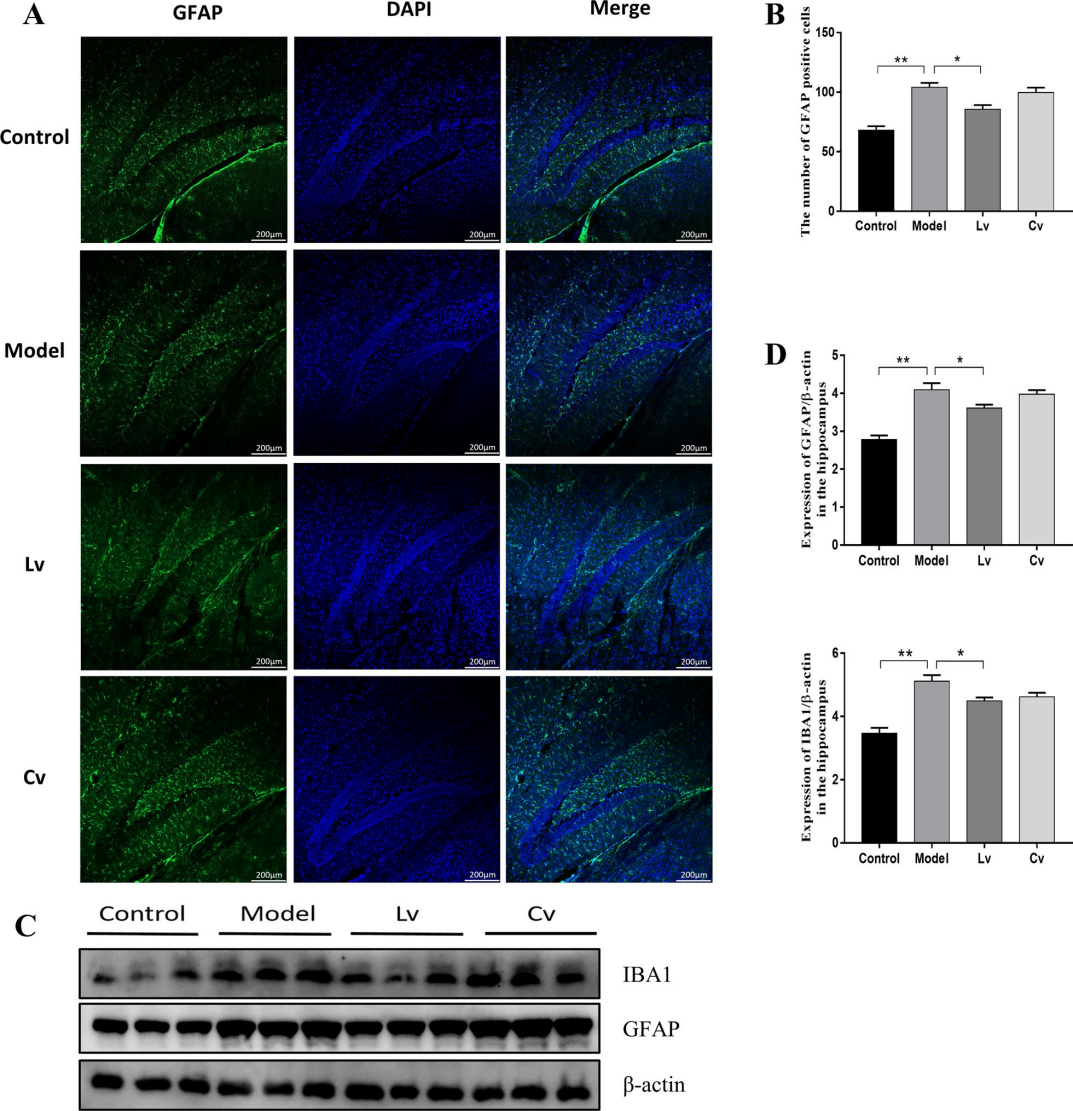
Lateral ventricle administration of BMSC-exos inhibited the activation of astrocytes in the hippocampus of mice injected with STZ. **A** Fluorescence detection of GFAP, a marker of activated astrocytes in hippocampus; **B** Quantification of the number of GFAP-positive cells. **C** Western Blot photos of IBA1 and GFAP; **D** IBA1 and GFAP protein expression levels quantification. Data are presented as means ± SEM, with *n* = 3 in each group (*P < 0.05, **P < 0.01)

Figures 5 and 6 show typical graphs and statistical analyses of the expression of IBA1 and GFAP in the hippocampus of mice. Compared with that of the control mice, the number of IBA1- and GFAP-positive cells and the protein expression of IBA1 and GFAP in the hippocampus of the model group were increased significantly, and these changes were reversed in the Lv group but not in the Cv group. As expected, the results of the Pearson test showed that the hippocampal expression of GFAP was positively correlated with the expression of IBA1 (*r* = 0.9311, *P* < 0.0001).

**Fig. 7:**
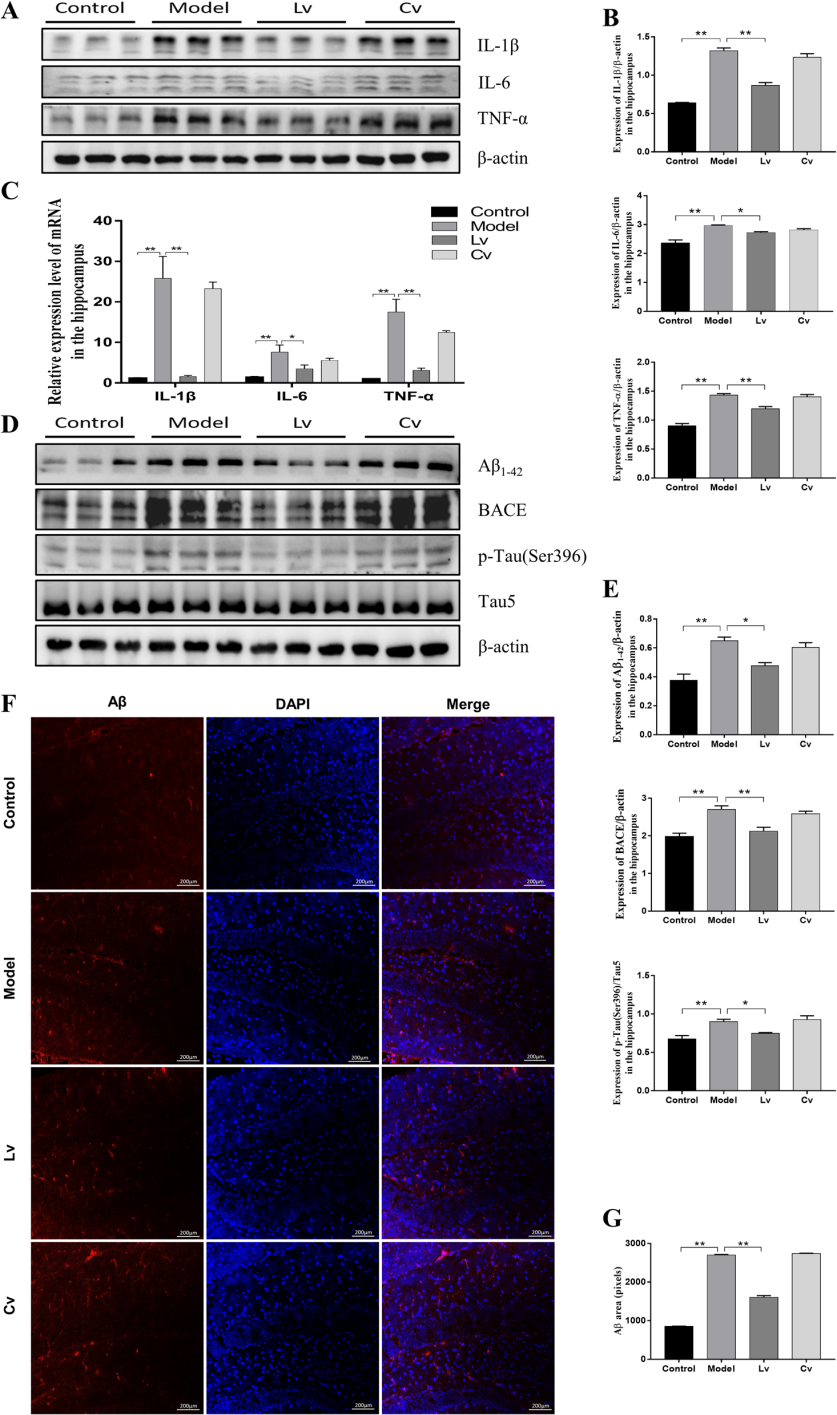
Lateral ventricle administration of BMSC-exos reduced the expression levels of IL-1β, IL-6 and TNF-α, Aβ, and p-Tau in the hippocampus of mice injected with STZ. **A** Typical Western Blot photos of IL-1β, IL-6 and TNF-α; **B** Quantitative analysis of the protein expression of IL-1β, IL-6 and TNF-α. **C** Quantitative analysis of the mRNA expression of IL-1β, IL-6 and TNF-α. **D** Typical Western blot photographs of Aβ_1-42_, BACE and p-Tau (ser396). **E** Quantitative analysis of the protein expression of Aβ_1-42_, BACE, and p-Tau (ser396) expression level. **F** Fluorescence detection of Aβ in hippocampus. **G** Quantification of the pixels of Aβ positive area. Data are presented as means ± SEM, with *n* = 3 in each group (*P < 0.05, **P < 0.01)

As shown in Fig. 7, the protein (Fig. 7A, B) and mRNA (Fig. 7C) expression levels of the inflammatory factors IL-1β, IL-6, and TNF-α in the hippocampus of the model mice were all remarkably increased compared with those of the control mice, which were reversed by treatment with BMSC-exos via lateral ventricle administration, but not caudal vein injection.

### Lateral ventricle administration, but not caudal vein injection of BMSC-exos, reduced amyloid accumulation and Tau hyperphosphorylation in the hippocampus of the STZ-injected AD mouse model

Compared with that of the control mice, the expression levels of BACE, Aβ_1-42_, and p-Tau (Ser396) in the hippocampus of the model group were significantly increased (Fig. 7D, E). There was no significant difference between the model and Cv groups, but the expression of these proteins was decreased significantly in the Lv group (Fig. 7D, E). The results of the IF experiment showed that the fluorescence intensity of Aβ_1-42_ in the hippocampus of the model mice was significantly stronger than that of the control mice. Compared with the model group, the hippocampal Aβ-positive and p-Tau-positive fluorescence intensity of the Lv group was significantly reduced, while there were no remarkable changes in the Cv group (Fig. 7F, G, Additional file 1, 2: Fig. S1, S2).

**Fig. 8:**
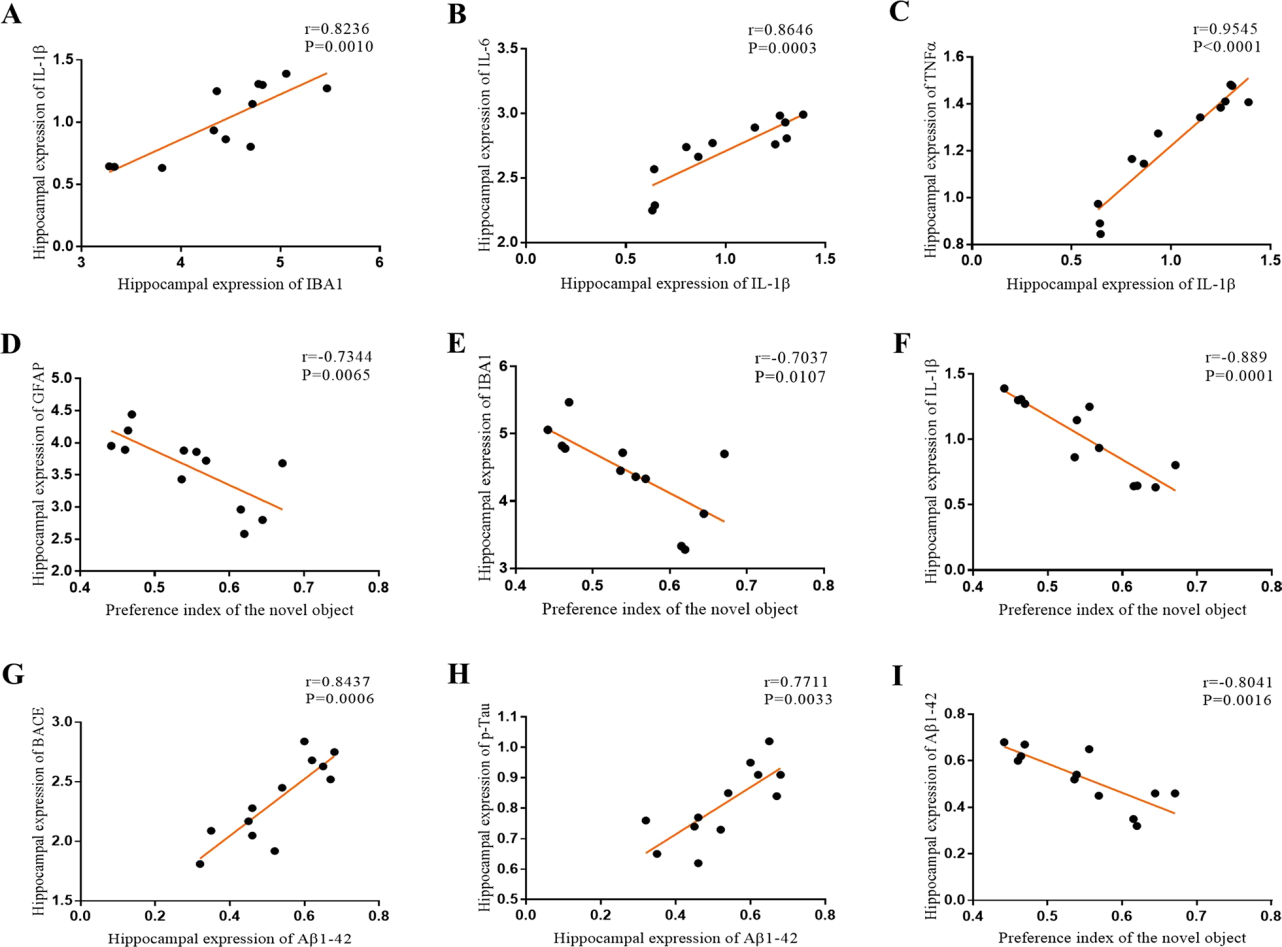
Correlation analysis between the hippocampal expression of GFAP, IBA1, IL-1β, IL-6, TNF-α, BACE, Aβ_1-42_, p-Tau and the preference index of novel object in the NOR. **A** Expression of IL-1β was positively correlated to IBA1. **B** Expression of IL-1β was positively correlated to IL-6. **C** Expression of IL-1β was positively correlated to TNFα. **D** The preference index of novel object was negatively correlated to the expression of GFAP. **E** The preference index of novel object was negatively correlated to expression of IBA1. **F** The preference index of novel object was negatively correlated to the expression of IL-1β. **G** Expression of BACE was positively correlated to Aβ_1-42_. **H** Expression of p-Tau was positively correlated to Aβ_1-42_. **I** Expression of Aβ_1-42_ was negatively correlated to the preference index of novel object

The results of Pearson's correlation test showed that the hippocampal expression of IL-1β was positively correlated not only with the hippocampal expression of IL-6 (*r* = 0.8646, *P* = 0.0003, Fig. 8B) and TNFα (*r* = 0.9545, *P* < 0.0001, Fig. 8C), but also with the expression of IBA1 (*r* = 0.8236, *P* = 0.0010, Fig. 8A). A positive correlation was also found between the hippocampal expression of Aβ_1-42_ and BACE (*r* = 0.8437, *P* = 0.0006, Fig. 8G) or p-Tau (*r* = 0.7711, *P* = 0.0033, Fig. 8H). Additionally, the preference index of the novel object in the NOR was negatively correlated with the expression of GFAP (*r* = − 0.7344, *P* = 0.0065, Fig. 8D), IBA1 (*r* = − 0.7037, *P* = 0.0107, Fig. 8E), IL-1β (*r* = − 0.889, *P* = 0.0001, Fig. 8F) and Aβ_1-42_ (*r* = − 0.8041, *P* = 0.0016, Fig. 8I) in the hippocampus.

### Lateral ventricle administration, but not caudal vein injection of BMSC-exos, promoted neuronal regeneration and the expression of BDNF in the hippocampus of the STZ-injected AD mouse model

**Fig. 9:**
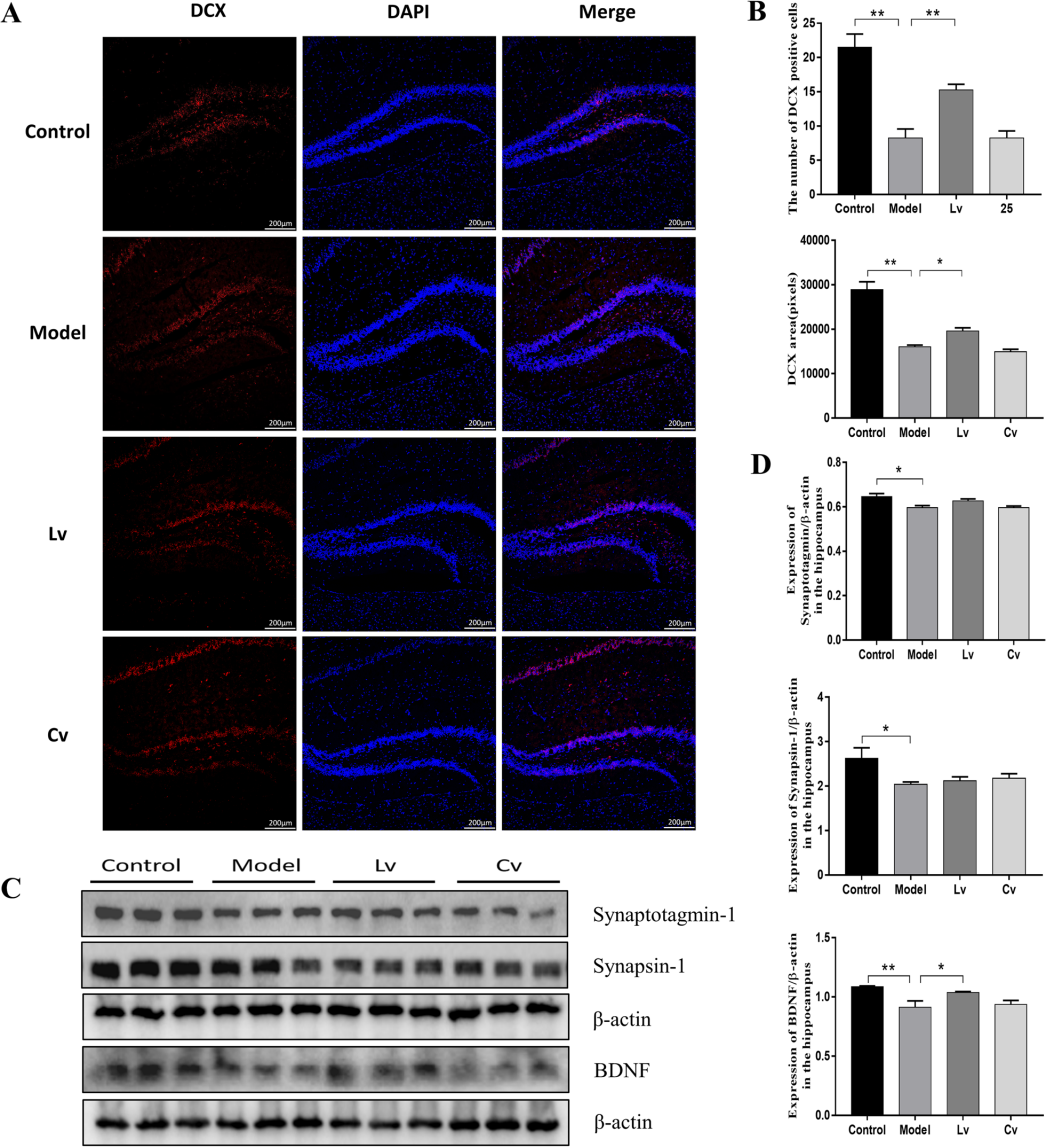
Lateral ventricle administration of BMSC-exos promoted neuron regeneration and the expression of BDNF in the hippocampus of mice injected with STZ. **A** Fluorescence detection of the hippocampal neonatal neuron marker DCX; **B** Statistics of the number of DCX positive cells and the quantification of the pixels of DCX positive area. **C** Western blot photos of Syt-1, Synapsin-1 and BDNF; **D** Quantification of Syt-1, Synapsin-1 and BDNF expression levels. Data are presented as means ± SEM, with *n* = 3 in each group (*P < 0.05, **P < 0.01)

Figure 9A, B shows the number of DCX-positive cells in the hippocampus of each group. Compared with the control group, the model mice had a reduced number of DCX-positive cells in the hippocampus. The number of DCX-positive cells in the Lv group was significantly higher than that in the model group, with no significant difference between the model group and the Cv group.

As shown in Fig. 9C, D, the expression levels of Syt-1, synapsin-1, and BDNF in the model group were significantly decreased as compared with that of the control group. Compared with the model group, the expression level of BDNF increased significantly in the Lv group, but not in the Cv group. The expression levels of Syt-1 and synapsin-1 were decreased in the model group, but did not change significantly after treatment with BMSC-exos, regardless of lateral ventricle administration or caudal vein injection.

**Fig. 10:**
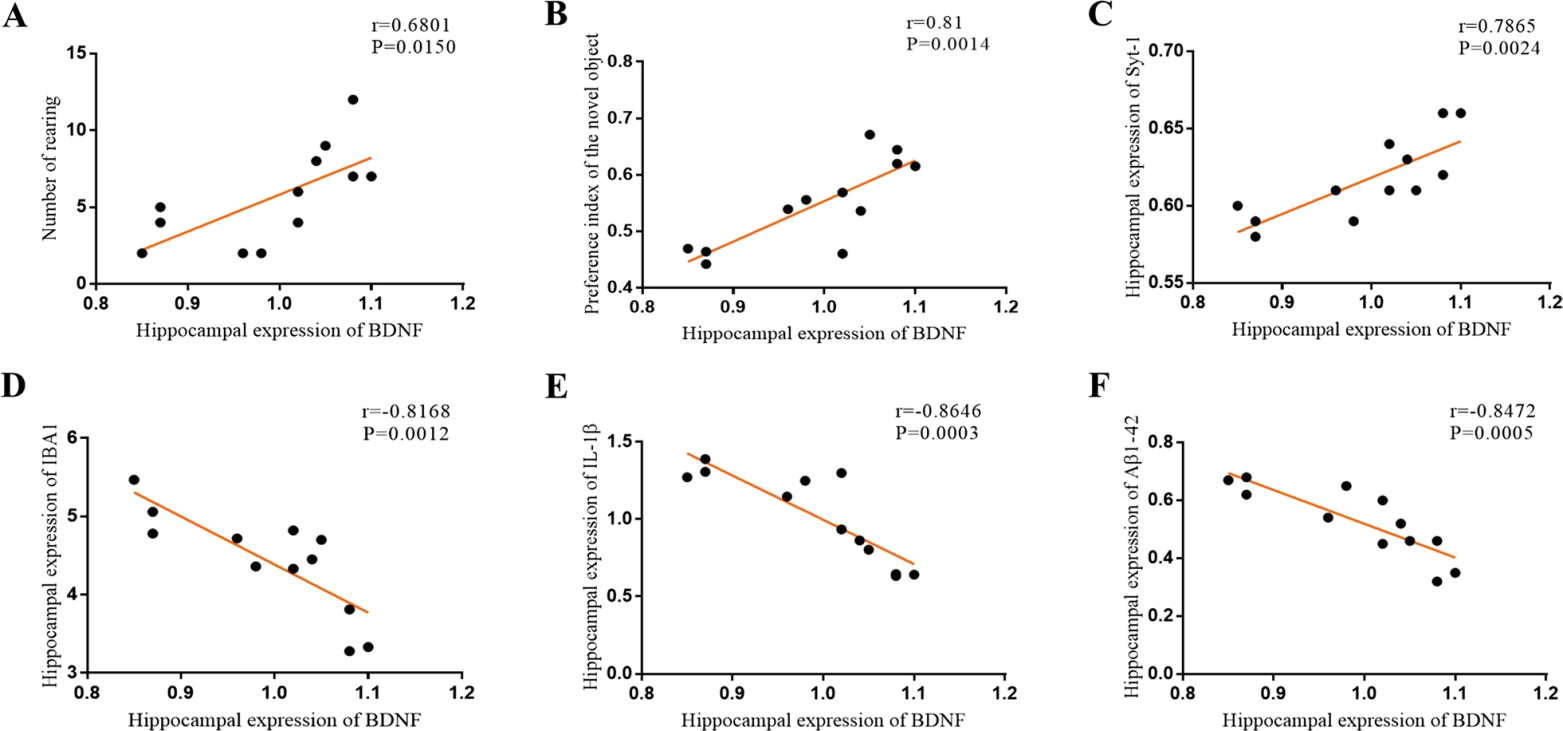
Pearson's correlation test between the hippocampus expression of GFAP, IBA1, IL-1β, IL-6, TNF-α, BACE, Aβ_1-42_, p-Tau, the number of rearing in the OFT and the preference index of novel object in the NOR. **A** The number of rearing was positively correlated to the expression of BDNF. **B** The preference of novel object was positively correlated to the expression of BDNF. **C** Expression of Syt-1 was positively correlated to BDNF. **D** Expression of IBA1 was positively correlated to BDNF. **E** Expression of IL-1β was positively correlated to BDNF. **F** Expression of Aβ_1-42_ was positively correlated to BDNF

The results of the Pearson's correlation test showed that the hippocampus expression of BDNF was positively correlated with the number of rearings in the OFT (*r* = 0.6801, *P* = 0.0150, Fig. 10A), the preference of novel object in the NOR (*r* = 0.81, *P* = 0.0014, Fig. 10B), and the hippocampus expression of synaptotagmin-1 (*r* = 0.7865, *P* = 0.0024, Fig. 10C), but negatively correlated with the protein expression of IBA1 (*r* = − 0.8168, *P* = 0.0012, Fig. 10D), IL-1β (*r* = − 0.8646, *P* = 0.0003, Fig. 10E), and Aβ_1-42_ in the hippocampus (*r* = − 0.8472, *P* = 0.0005, Fig. 10F).

## Discussion

In the present study, we investigated the potential effect of BMSC-exos on AD-like behaviors in an STZ-injected mouse model, and the results showed that lateral ventricle administration of BMSC-exos could improve AD-like behaviors, as indicated by the increased motion and rearing in the OFT, together with an increased preference index of novel objects in the NOR. Moreover, hippocampal inflammation and hyperactivation of glial cells in the AD mouse model were significantly improved after lateral ventricle administration of BMSC-exos, and the protein expression levels of BACE, Aβ_1-42_ and p-Tau were significantly reduced, while the expression level of BDNF was significantly increased. Furthermore, the results of the Pearson test showed that the preference index of the novel object in the NOR was positively correlated with the hippocampal expression of BDNF but negatively correlated with the expression of GFAP, IBA1, and IL-1β. Apart from a positive correlation between the hippocampal expression of BDNF and Syt-1, BDNF abundance was found to be negatively correlated with markers of glial activation and the expression of the inflammatory cytokines, Aβ, and p-Tau, which are characteristic neuropathological features of AD. These results indicated that lateral ventricle administration, but not caudal vein injection of BMSC-exos, can improve AD-like behaviors in STZ-injected mice, the mechanism of which might be involved with the regulation of glial activation and its associated neuroinflammation, together with BDNF-related neuropathological changes in the hippocampus.

STZ is a glucosamine–nitrosourea and DNA alkylating reagent that synthesized by *Streptomycetes achromogenes*. STZ could result in insulin resistance and has been widely used to establish animal models of diabetes and AD [31]. It has been reported that lateral ventricle injection of STZ could induce AD-like behavior in rats [32] or mice [33–37], as indicated by significant cognitive impairment and increased Aβ_1-42_ accumulation and hyperphosphorylated tau in the hippocampus [32], along with increased glial activation or decreased expression of synapse-associated protein [33–37]. In our previous study, STZ was administered via bilateral hippocampal injection, and the mice displayed impaired learning and memory abilities, together with insulin resistance and hyperactivated microglial and alternative synaptic plasticity in the hippocampus [38]. Consistently, in the present study, our results showed that lateral ventricle injection of STZ mice could induce AD-like behavioral performance in mice, as indicated by the decreased frequency, duration, and distance traveled in the center area and reduced rearing frequency in the OFT, increased immobility in the TST, and the decreased preference index of novel object or arm in the NOR or Y-maze. As expected, a positive correlation was found between decreased activity in the OFT and decreased learning and memory ability in the NOR. These results again indicated that STZ was an effective reagent in establishing AD-like animal models. Moreover, in the present study, mice treated with BMSC-exos via lateral ventricle injection, but not caudal vein injection, presented increased activity and enhanced rearing frequency in the OFT, together with an increased preference index of novel objects in the NOR. These results indicated that lateral ventricle injection of BMSC-exos could reverse STZ-injected AD-like behaviors in mice in a task-specific manner. We could not interpret exactly the reason for the different effects of BMSC-exos between the two delivery routes, although the IF results showed that BMSC-exos labeled with PKH26 could be detected in the hippocampus when administered by lateral ventricle injection but not caudal vein injection. It has been reported that exosomes can cross the blood–brain barrier (BBB) [39–41]. However, there is still controversy about the ability of exosomes to reach the brain via peripheral circulation, suggesting the opinion that the biodistribution of extracellular vesicle is determined by the cell source, route of administration, and targeting [42]. Focusing on the absorption and distribution dynamics of exosomes*,* it has also been reported that most of the exosomes injected via tail vein reached the liver, kidney, and other peripheral organs in the body, and the amount of exosomes that entered the brain was relatively small [42–44]. Additionally, the discrepancy in the present study might be, at least partly, ascribed to the limited injection volume.

The activation and proliferation of central glial cells is one of the most significant pathological features of AD. Microglia are considered to be the resident macrophages of the central system, which are in close contact with neurons and astrocytes [45, 46]. Microglia play two roles in the entire pathological process of AD, by acting as the "cleaner" in the early stage of AD to the "accomplice" in the later stage [47]. From a morphological point of view, round microglia have a phagocytic function, and studies have confirmed this finding. The microglia in the drug treatment group recover from the activated irregular polygon shape to a round shape, and the Aβ contained in their phagosomes is twice that of activated microglia [48]. After the body is injured, microglia take the lead to respond, and then astrocytes begin to respond [49]. After activation, astrocytes release inflammatory factors, similar to activated microglia, which further aggravates the neuroinflammation. In our present study, the number of activated microglia and astrocytes in the hippocampus of the model group mice increased significantly. After treatment with BMSC-exos, the number of activated microglia and astrocytes decreased significantly in the Lv group, but not in the Cv group, and the number of round microglia in the Lv group was also increased significantly and returned to normal levels in comparison with the model group. Moreover, the results of the Western blot was similar to the IF results, and the expression of GFAP and IBA1 was positively correlated. These results again indicated the important role of glial activation in the pathogenesis of AD, and it might be a potential target of BMSC-exos in relieving AD-like performance.

As mentioned earlier, the activation of glial cells strengthens the inflammatory response, and the interaction between glial cells and inflammatory factors forms a vicious cycle and can trigger a series of malignant processes, such as promoting the accumulation of Aβ and phosphorylation of Tau protein [50–54], which are two characteristic neuropathological hallmarks of AD. Consistently, in the present study, the mRNA and protein expression levels of IL-1β, IL-6, and TNF-α in the hippocampus of the model mice were upregulated, together with increased protein expression of BACE, Aβ_1-42,_ and p-Tau. Moreover, apart from the expected positive correlation between inflammatory cytokines, the expression of IL-1β was found to be positively correlated with the expression of GFAP, IBA1, BACE, Aβ_1-42_ and p-Tau. Additionally, the preference index of the novel object in the NOR was negatively correlated with the neuroinflammation markers in the hippocampus. These findings again suggested the network hypothesis of AD, which proposes that AD is a complex and multifactorial disease with the compromised functionality of relevant neural networks underlying the development of AD symptomatology. After intracerebroventricular injection of BMSC-exos, the levels of inflammatory factors were significantly downregulated, and the protein expression levels of BACE, Aβ_1-42_, and p-Tau were also significantly reduced. These results indicated that BMSC-exos may alleviate central nervous system inflammation and improve the typical neuropathological features of AD in mice injected with STZ.

Synaptic plasticity is a crucial basis for regulating learning and memory functions [38]. It has long been demonstrated that the expression levels of synapse-related proteins such as Syt-1 and synapsin-1 are significantly reduced in the hippocampal tissues of postmortem AD patients and animal models [33, 37, 55, 56]. BDNF is one of the important regulators that underlies plasticity [57] and can provide nutritional support for neurons and improve learning and memory impairment by reversing neuronal loss [58]. In our previous studies, an imbalance in the expression of BDNF in the hippocampus or prefrontal cortex and its related adaptive change in synaptic plasticity, including decreased expression of Syt-1, synapsin-1, and PSD95 has been associated with impaired learning and memory or depression-like behaviors in animal models with depression [59], nonalcoholic fatty liver disease (NAFLD) [60] or AD [38]. In the field of exosomes, it has been reported that the BDNF/TrkB/CREB pathway was activated following treatment with miR-206 modified exosomes isolated from human umbilical cord mesenchymal stem cells (hucMSC) in a subarachnoid hemorrhage rat model [61], and enhancing BDNF level and balancing inflammatory response have been reported to be involved in the mechanism underlying the analgesic effects of hucMSC-derived exosomes [62]. Moreover, exosomes extracted from BDNF-pretreated MSCs could effectively promote functional recovery and neurogenesis of rats after traumatic brain injury, the mechanism may be related to the high expression of miR-216a-5p [63], and exosomes derived from BDNF-overexpressing 293 T cells were reported to have a stronger neuroprotective effect than that from cells without overexpression of BDNF [64, 65]. In the present study, the expression of BDNF, Syt-1, and synapsin-1 was decreased in the hippocampus of STZ-intracerebroventricular injected mice, again suggesting the important role of BDNF and its associated synaptic proteins in the maintenance of learning and memory. Together with its significant correlation with the behavioral performance, inflammatory response, and other AD-like neuropathological features, our results indicated that BDNF might be a key molecule linking the relevant multiple pathogenic mechanisms the development of AD. However, the decreased expression of BDNF in the AD mouse model was reversed by lateral ventricle administration of BMSC-exos. Although there was no significant difference between mice in the model group and the Lv group, the IF results showed that the expression of DCX, a marker of newborn neurons, was significantly increased in the hippocampus of mice in the Lv group compared with that in the model group. These results suggested that BMSC-exos may regulate the prominent plasticity of the hippocampus by upregulating the expression of BDNF and promoting the neuronal regeneration.

## Conclusions

**Fig. 11:**
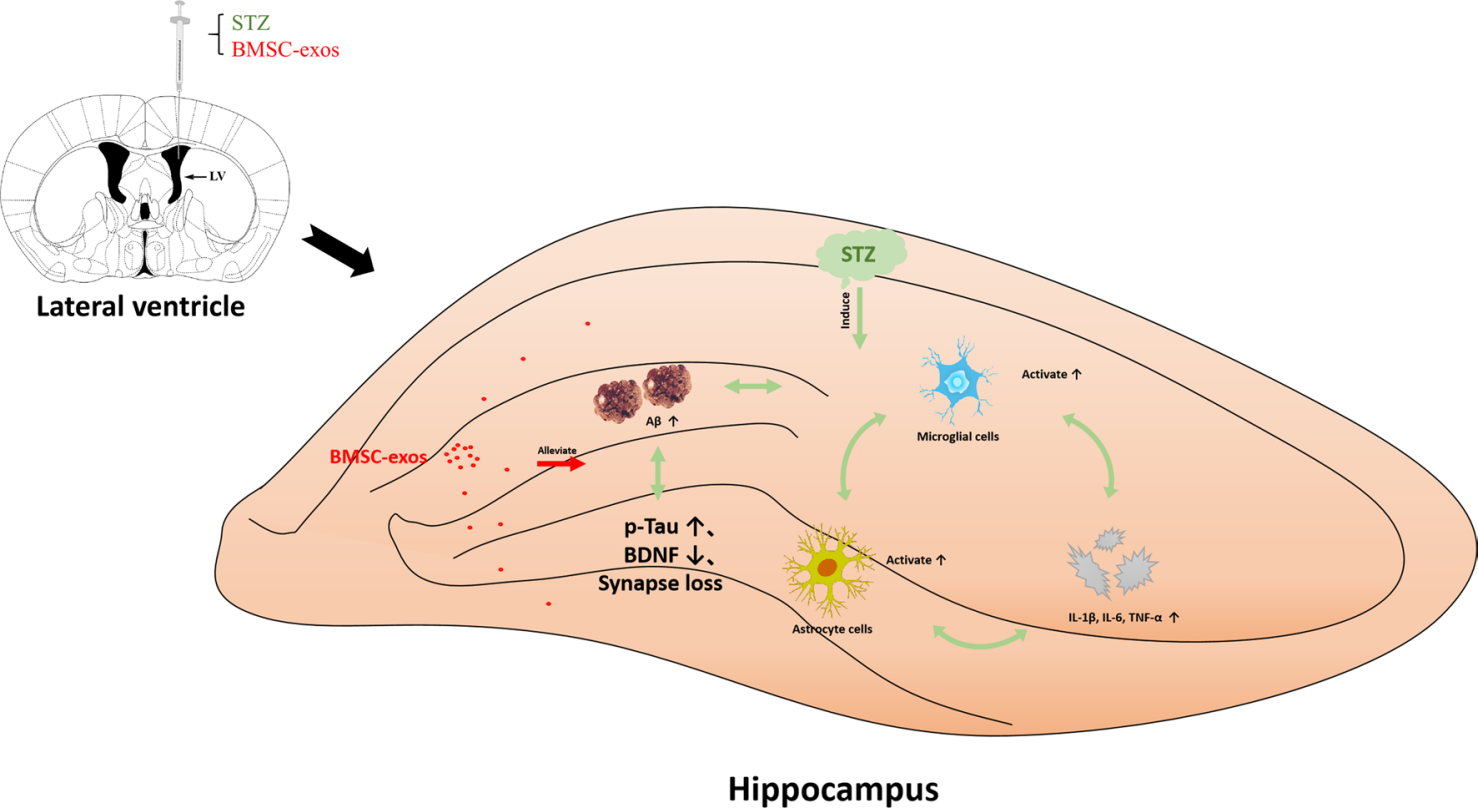
Exosomes derived from bone-marrow mesenchymal stem cells allivate neuroinflammation and synaptic damage by inhibiting the activation of glial cells in the hippocampus

In conclusion, our results indicated that lateral ventricle administration of BMSC-exos could improve AD-like behaviors in mice, the mechanism of which might be associated with the regulation of glial activation and its associated neuroinflammation and BDNF-related neuropathological changes in the hippocampus. The summary diagram is shown in Fig. 11.

## Supplementary Information

**Additional file 1: Fig. S1.** Lateral ventricle administration of BMSC-exos reduced the expression levels of Aβ in the hippocampus of mice injected with STZ. Typical photos of Thioflavin S staining in each group of hippocampus. (*n* = 3 in each group). **Fig. S2.** Lateral ventricle administration of BMSC-exos reduced the expression levels of p-Tau in the hippocampus of mice injected with STZ. A: Fluorescence detection of p-Tau in hippocampus. B: Quantification of the pixels of p-Tau positive area. Data are presented as means ± SEM, with *n* = 3 in each group (*P < 0.05, **P < 0.01).

**Additional file 2: Fig. S2.** Lateral ventricle administration of BMSC-exos reduced the expression levels of p-Tau in the hippocampus of mice injected with STZ. A: Fluorescence detection of p-Tau in hippocampus. B: Quantification of the pixels of p-Tau positive area. Data are presented as means ± SEM, with *n* = 3 in each group (*P < 0.05, **P < 0.01).

## Data Availability

The data sets used and/or analyzed in the current study are available from the corresponding author on reasonable request.

## Abbreviations

AD: Alzheimer's disease
Aβ: β-Amyloid
ACHE: Acetylcholin esterase
BDNF: Brain-derived neurotrophic factor
BMSCs: Bone-marrow mesenchymal stem cells
BMSC-exos: Exosomes derived from bone-marrow mesenchymal stem cells
CD11b: Cluster of differentiation 11b
CD44: Cluster of differentiation 44
CD45: Cluster of differentiation 45
CD63: Cluster of differentiation 63
DCX: Recombinant Doublecortin
EPM: Elevated plus maze test
FDA: U.S. Food and Drug Administration
GFAP: Glial fibrillary acidic protein
HSP70: Heat shock protein 70
IBA1: Ionized calcium binding adapter molecule 1
IF: Immunofluorescence staining
IL-1β: Interleukin 1β
IL-6: Interleukin 6
miRNAs: MicroRNAs
MPC5: Mouse renal podocytes
NGF: Nerve growth factor
NOR: Novel object recognition test
OFT: Open field test
p-Tau: Phosphorylated Tau protein
qPCR: Quantitative polymerase chain reaction
STZ: Streptozotocin
Syt-1: Synaptotagmin-1
TNF-α: Tumor necrosis factor
TSG101: Tumor susceptibility gene 101 protein
TST: Tail suspension test
Y-maze: Y maze test
