# Supplementary figures and images for: Exosomes derived from bone-marrow mesenchymal stem cells alleviate cognitive decline in AD-like mice by improving BDNF-related neuropathology
Source: J Neuroinflammation. 2022 Feb 7;19:35. doi: 10.1186/s12974-022-02393-2 (PMC8822863; doi:10.1186/s12974-022-02393-2)

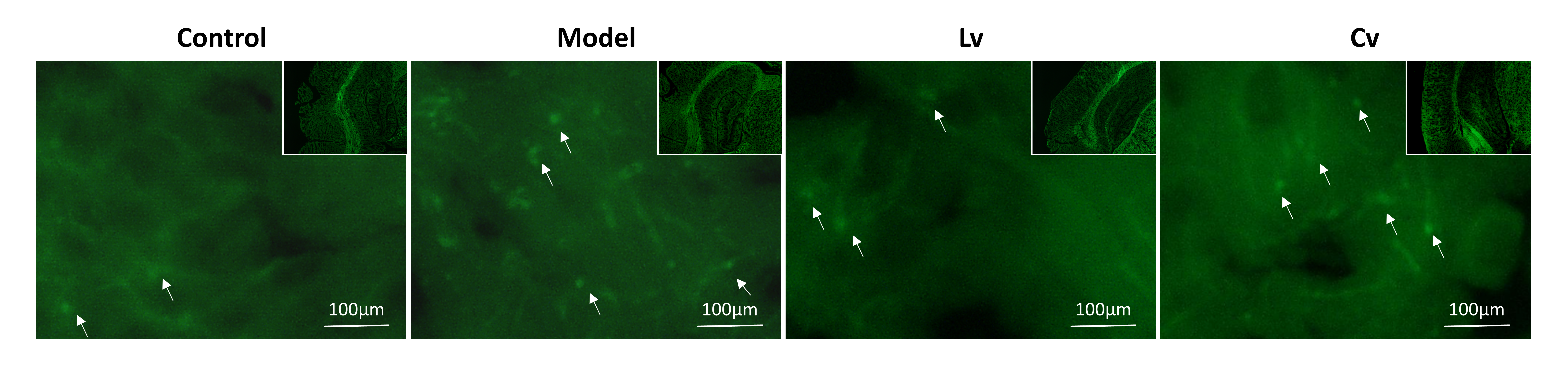

Supplement: Supplementary file 1 — Additional file 1: Fig. S1. Lateral ventricle administration of BMSC-exos reduced the expression levels of Aβ in the hippocampus of mice injected with STZ. Typical photos of Thioflavin S staining in each group of hippocampus. (n = 3 in each group). Fig. S2. Lateral ventricle administration of BMSC-exos reduced the expression levels of p-Tau in the hippocampus of mice injected with STZ. A: Fluorescence detection of p-Tau in hippocampus. B: Quantification of the pixels of p-Tau positive area. Data are presented as means ± SEM, with n = 3 in each group (*P < 0.05, **P < 0.01). [file 12974_2022_2393_MOESM1_ESM.tif]

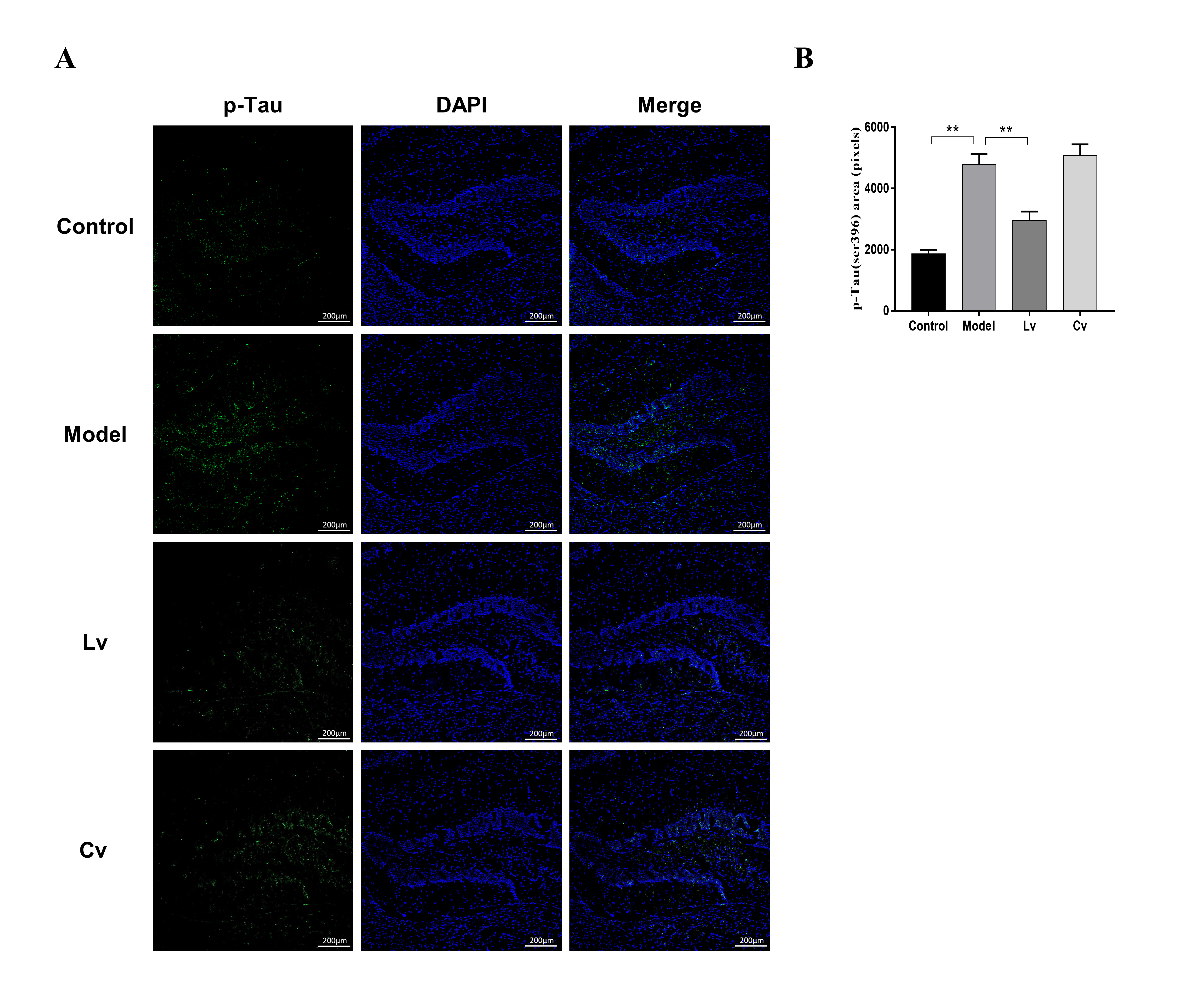

Supplement: Supplementary file 2 — Additional file 2: Fig. S2. Lateral ventricle administration of BMSC-exos reduced the expression levels of p-Tau in the hippocampus of mice injected with STZ. A: Fluorescence detection of p-Tau in hippocampus. B: Quantification of the pixels of p-Tau positive area. Data are presented as means ± SEM, with n = 3 in each group (*P < 0.05, **P < 0.01). [file 12974_2022_2393_MOESM2_ESM.tif]
